# Ceftriaxone-by-default in surgical prophylaxis across the East African community: a measurement problem

**DOI:** 10.3389/frabi.2026.1968363

**Published:** 2026-10-12

**Authors:** Samar Rashid Abdullahi, Nathan Mugenyi, Lucy Ochola, Jamal Abade Mohamed, Hussein Iman Sigat, Abdirizak Ali Yusuf, Wigilya P. Mikomangwa, Armstrong Ndihokubwayo, David Majuch Kunjok, Schifra Uwamungu, Nathan Banzi, Barbra Tuhamize

**Affiliations:** 1 Human Health Department, One Health Antimicrobial Resistance Network (OHAAN), Nairobi, Kenya; 2 Medical Department, Wajir County Referral Hospital, Wajir, Kenya; 3 Department of Community Health, Faculty of Medicine, Mbarara University of Science and Technology, Mbarara, Uganda; 4 Surgical Department, Garissa County Referral Hospital, Garissa, Kenya; 5 Infectious Disease Department, National Institute of Health, Mogadishu, Somalia; 6 Department of Clinical Pharmacy and Pharmacology, School of Pharmacy, Muhimbili University of Health and Allied Sciences, Dar es Salaam, Tanzania; 7 National Reference Laboratory, National Institute of Public Health, Bujumbura, Burundi; 8 Department of Environmental Health and Disease Control, School of Public Health, Jomo Kenyatta University of Agriculture and Technology, Nairobi, Kenya; 9 Expanded Programme on Immunization (EPI), Ministry of Health, Juba, South Sudan; 10 Biomedical Laboratory Sciences Department, School of Health Sciences, College of Medicine and Health Sciences, University of Rwanda, Kigali, Rwanda; 11 Department of Microbiology and Parasitology, Faculty of Health Sciences, Mbarara University of Science and Technology, Mbarara, Uganda; 12 South African Medical Research Council–University of Venda Antimicrobial Resistance and Global Health Research Unit (SAMRC-UNIVEN) Antimicrobial Resistance and Global Health Research Unit, Institute for Pathogen and Global Health Research, University of Venda, Thohoyandou, South Africa; 13 Department of Science, Technology and Innovation (DSTI) / National Research Foundation (NRF) South African Research Chairs Initiative (SARChI), Institute for Pathogen and Global Health Research, University of Venda, Thohoyandou, South Africa

**Keywords:** antimicrobial consumption surveillance, antimicrobial stewardship, cefazolin, ceftriaxone, cephalosporin resistance, East African community, surgical antibiotic prophylaxis

## Abstract

Surgical antibiotic prophylaxis across the East African Community is dominated by ceftriaxone, a WHO Access, Watch, and Reserve (AWaRe) Watch-group third-generation cephalosporin not recommended for routine prophylaxis. The practice recurs across regional point prevalence surveys, yet consumption surveillance cannot distinguish prophylactic from therapeutic use. Drawing on antimicrobial-use surveys, surgical-prophylaxis studies, resistance surveillance, and medicine-availability reports, we argue that four interacting failure modes sustain it: substitution of first-line agents, prolongation of postoperative prophylaxis, unnecessary combination, and empirical spillover from ward prescribing. Resistance among surgical-site isolates is already high enough to threaten the value of prophylaxis itself. Prophylaxis is measured for duration, documented indication, and guideline concordance, and agent choice is captured in point-prevalence surveys, but none of this reaches the national consumption totals that govern procurement, and no indicator records the supply constraint determining the agent or the resistance it selects. We propose four measures, one requiring routing rather than building and three genuinely absent, that would convert an ungoverned practice into a governable one.

## 1 Introduction

Surgical prophylaxis is among the most dependable interventions in contemporary surgery. A single dose of a narrow-spectrum agent, administered within 60 min of incision and stopped at wound closure, prevents a substantial share of surgical-site infections (SSI) at negligible ecological cost. WHO guidelines and international surgical societies are consistent on three points: a narrow-spectrum agent, typically cefazolin; one pre-incision dose; discontinuation at closure (Bratzler et al., 2013). Extending prophylaxis beyond closure adds no further reduction in SSI while increasing consumption and selective pressure (de Jonge et al., 2020; World Health Organization, 2018). The WHO Access, Watch, and Reserve (AWaRe) classification places cefazolin in the Access group; ceftriaxone is a Watch agent whose use is to be monitored for its greater resistance-selection potential (World Health Organization, 2023).

Practice across the East African Community (EAC) departs from that guidance at every step, for reasons as much structural as behavioural. Where cefazolin is intermittently stocked or absent from procurement lists, ceftriaxone becomes the most accessible injectable cephalosporin and the effective default (Alemkere et al., 2023). A woman delivering by caesarean section at a regional referral hospital in Kakamega, Mbarara, Dar es Salaam, or Kigali typically receives intravenous ceftriaxone before incision, usually with metronidazole, and again daily for several days afterwards. No specimen is cultured. The choice of agent reflects no clinical decision because none was required: ceftriaxone is what the pharmacy reliably holds, what the senior surgeon expects, and what the ward routine endorses.

The cumulative effect on cephalosporin resistance is unquantified. A contribution of ceftriaxone exposure to selection for cephalosporin resistance is biologically plausible based on its pharmacological properties, and the high levels of resistance reported among surgical isolates are consistent with this possibility. However, the magnitude of this contribution, and the proportion attributable specifically to prophylaxis, have not been measured (Lester et al., 2020). What keeps it unquantified is not absent observation but absent measurement: the practice is named in every point-prevalence survey yet appears in no national indicator, because consumption surveillance aggregates ceftriaxone across all indications.

This perspective sets out why the agent matters, what the region's surgical isolates show, why existing instruments cannot resolve the exposure that produced them, and what a minimum measurement standard for the eight Partner States should contain. The region does not need new stewardship principles; it needs improved measurement of an exposure that is currently difficult to distinguish from therapeutic use.

## 2 The regional case

### 2.1 The agent

The objection to ceftriaxone rests on the pharmacology of the molecule rather than a general preference for narrow spectra. Cefazolin has a half-life of about 2 h, penetrates most surgical sites well, and covers the organisms responsible for most clean and clean-contaminated procedures, which underpins its recommendation as the preferred first-line prophylactic agent (Bratzler et al., 2013; World Health Organization, 2018). Its effect on colonic flora is correspondingly limited. Ceftriaxone differs on both axes that determine selection: its long half-life sustains sub-inhibitory exposure well beyond the operative window, and a substantial fraction of each dose is excreted in bile into the gut lumen, acting on the densest reservoir of Enterobacterales in the body (Alasmari et al., 2023; Arvidsson et al., 1982). Exposure delivered straight to colonic flora efficiently selects for extended-spectrum β-lactamase (ESBL)-producing *E. coli* and *K. pneumoniae*, the organisms that subsequently cause resistant surgical-site and bloodstream infections (Arvidsson et al., 1982).

The pharmacological rationale for preferring cefazolin does not depend on having a regional estimate of the contribution of prophylactic ceftriaxone to resistance. However, the absence of such an estimate limits the ability to quantify this contribution. Measurement is therefore needed to determine the extent of the problem and to assess the potential impact of interventions.

### 2.2 Four modes of failure

Standard stewardship audit decomposes inappropriate prophylaxis into indication, agent selection, dosing, timing, and duration (Nathwani et al., 2019). Those categories assess concordance with a guideline; they do not ask why a single molecule dominates. The four modes below are organised differently: each is a mechanism producing the ceftriaxone default, each carries a distinct measurable signal, and each sits with a different actor.

#### 2.2.1 Substitution

Ceftriaxone is used where cefazolin is indicated, most often because cefazolin is unavailable. The mechanism has been documented in Ethiopia, where cefazolin is the first-line prophylactic agent in the national Standard Treatment Guideline and appears on the national Essential Medicines List, yet was unavailable for years and was removed from the government procurement list despite both recommendations (Alemkere et al., 2023); stewardship teams there reverted to ceftriaxone once cefazolin was out of stock. The commercial logic generalises: where no clear demand signal or supporting policy exists, suppliers favour broad-spectrum agents with multiple uses over a narrow agent with a single purpose, and thin margins on old injectable generics give manufacturers little reason to expand capacity (Alemkere et al., 2023). Across several EAC settings, cefazolin has been reported as intermittently available while ceftriaxone is more consistently stocked, although the extent of this pattern has not been quantified regionally. Cefazolin is an Access-group agent and an off-patent generic; the available evidence suggests that supply constraints may contribute to its underuse. The default is therefore potentially influenced by procurement and supply-chain constraints and prescriber behaviour.

#### 2.2.2 Prolongation

A single pre-incision dose becomes a multi-day postoperative course. This is the most consequential mode for selection, because prolonged exposure increases antimicrobial consumption and may increase selective pressure on gut flora, although the contribution of prophylaxis duration to regional resistance has not been directly quantified. At the University Teaching Hospital of Kigali and at Kakamega County Teaching and Referral Hospital, prophylaxis exceeded 1 day in over 83% of surgical patients (Igizeneza et al., 2024; Sifuna et al., 2026); across three hospitals in north-eastern Tanzania, it continued beyond 3 days in 57% of cases, with ceftriaxone the commonest agent (Horumpende et al., 2020). In Uganda, only 4% of surgical patients at a regional referral hospital completed prophylaxis on the day of surgery (mean 3.5 days; ceftriaxone plus metronidazole in 67.1% of prescriptions) (Saito et al., 2020), and at Kawempe National Referral Hospital a 2–3-day postoperative course after caesarean section is hospital policy (Mbabazi et al., 2025). These findings indicate that prolonged prophylaxis occurs in multiple settings, although they should not be interpreted as a representative estimate for the EAC region.

#### 2.2.3 Combination

Ceftriaxone is co-prescribed with metronidazole for caesarean and abdominal surgery, frequently without a documented anaerobic indication; in the Kawempe series, anaerobes were not among the organisms recovered (Mbabazi et al., 2025). Anaerobic cover has a rationale in contaminated and some clean-contaminated surgery; the objection is not to metronidazole where indicated but to its routine addition irrespective of procedure class (Bratzler et al., 2013).

#### 2.2.4 Empirical spillover

Ceftriaxone, the workhorse of undifferentiated sepsis on the wards, becomes the reflex in theatre, potentially allowing patterns of therapeutic prescribing to influence prophylactic choice. Ugandan surveys show ceftriaxone accounting for 37% of antibiotic prescriptions across 13 hospitals and being the most frequently prescribed Watch agent for both therapeutic and prophylactic indications, with guideline compliance of 30% (Kiggundu et al., 2022); across nine regional referral hospitals, 24.1% of prescriptions lacked a documented indication, and four agents accounted for over three-quarters of use (Nakasendwa et al., 2026). In Tanzania, 90% of surgical prophylaxis prescriptions carried no documented justification (Schrama et al., 2025). Limited documentation makes it difficult to determine the extent to which this reflects clinical indication, institutional practice, or empirical prescribing. These modes are layers, not alternatives: one caesarean episode can substitute ceftriaxone for cefazolin, combine it with metronidazole, and prolong it for 5 days.

### 2.3 What the wound isolates show

Where indication-level prescribing data are absent, resistance data are increasingly available (**Table 1**). Third-generation cephalosporin (3GC) resistance among Enterobacterales cultured from surgical-site infection runs from roughly three-quarters to unanimity. In post-caesarean SSI at Mulago, Kampala, all 11 *E. coli* and 43 of 44 *Klebsiella* isolates were ceftriaxone-resistant (Wekesa et al., 2020); at Kawempe the dominant gram-negatives were 94%–100% 3GC-resistant (Mbabazi et al., 2025); at Kirehe, Rwanda, 92.1% of post-caesarean isolates were ceftriaxone-intermediate or resistant (Velin et al., 2021). A systematic review and meta-analysis of 59 studies across Central, Eastern, Southern, and Western Africa found resistance in *E. coli* above 40% for every antibiotic class except carbapenems, and in *K. pneumoniae* above 30% for every class except carbapenems (Monk et al., 2024). Its authors noted that 3GC resistance in *E. coli* exceeded what global burden estimates predicted, whereas resistance in *K. pneumoniae* was consistent with them, and suggested this might reflect the proportion of surgical-site infections in the review and the use of ceftriaxone in surgical prophylaxis selecting out 3GC-resistant isolates (Monk et al., 2024). Nor is the problem confined to acute wounds: infected chronic wounds on surgical wards in south-western Uganda show substantial 3GC resistance (Wangoye et al., 2022). These findings demonstrate a high prevalence of resistance among several groups of surgical and wound isolates, but they do not establish that prophylactic ceftriaxone exposure caused or substantially contributed to this resistance.

**Table 1:** Ceftriaxone resistance in surgical-site and related clinical isolates, East African community partner states.

Partner state	Setting; procedure	Isolate type	Ceftriaxone or ESBL finding	Source
Uganda	Mulago, Kampala; caesarean SSI	SSI	*E. coli* 100% (11/11); *Klebsiella* 97.7% (43/44); ESBL explained ceftazidime resistance in 10/11 *E. coli*	(Wekesa et al., 2020)
Uganda	Kawempe, Kampala; post-caesarean SSI	SSI	Dominant gram-negatives 94–100% 3GC-resistant; carbapenem-resistant *Acinetobacter* 65.4%	(Mbabazi et al., 2025)
Uganda	Mbarara RRH; SSI (n = 83)	SSI	96.7% of gram-negative isolates multidrug-resistant; high ceftriaxone resistance among gram-negatives	(Hope et al., 2019)
Tanzania	Muhimbili, Dar es Salaam; SSI	SSI	ESBL 79.3% of Enterobacteriaceae (*E. coli* 92.3%)	(Manyahi et al., 2014)
Tanzania	Tertiary hospitals, Dar es Salaam; SSI	SSI	Gram-negative bacteria 76.8% of SSI; ceftriaxone resistance above 40%	(Chipaga et al., 2025)
Rwanda	Kirehe DH; post-caesarean SSI	SSI	92.1% ceftriaxone-intermediate or resistant	(Velin et al., 2021)
Kenya	Kisii TRH, surgical unit; wounds (mixed)	Wound (mixed)	Ceftriaxone resistance 33% across wound isolates; mixed wound infections, not SSI-specific	(Onsando et al., 2025)
Kenya	National referral and level-5; clinical isolates including wound	Clinical (mixed)	*E. coli* 69.6%; *K. pneumoniae* 91.3% ceftriaxone-resistant (two-facility clinical series)	(Maveke et al., 2024)
Burundi; DR Congo	MSF obstetric sites; post-caesarean SSI	None cultured	SSI incidence 7.3%; no organisms cultured	(Chu et al., 2015)
Somalia	Mogadishu; clinical isolates, not disaggregated	Clinical (mixed)	High resistance across Enterobacterales; not SSI-specific	(Ibrahim et al., 2025)
South Sudan	Not applicable	Not applicable	No surgical-site resistance data identified	Not applicable
Region	59 studies; wound, SSTI, and SSI, pooled	Wound/SSTI/SSI	*E. coli* above 40% resistant to all classes except carbapenems (6%); *K. pneumoniae* above 30% except carbapenems (8%)	(Monk et al., 2024)

Chu et al. (2015): DR Congo pooled MSF multi-country figure; Sierra Leone included.

SSI, surgical-site infection; 3GC, third-generation cephalosporin; ESBL, extended-spectrum β-lactamase; RRH, regional referral hospital; TRH, teaching and referral hospital; DH, district hospital; MSF, Médecins Sans Frontières; SSTI, skin and soft-tissue infection.

These series are heterogeneous, differing in case definition, specimen type, and setting; several are not confined to SSI, several rest on small denominators, and all come from referral centres where resistant organisms concentrate. Together, they provide evidence of substantial resistance in the reported settings, but not a standardised regional rate or a causal estimate of the contribution of prophylactic ceftriaxone.

Where most Enterobacterales causing SSI are already ceftriaxone-resistant, the effectiveness of ceftriaxone as prophylaxis against these organisms may be limited. However, these data cannot determine whether prophylactic ceftriaxone exposure contributed to the observed resistance. They are drawn from infections that occurred, not from the flora of patients administered prophylaxis. They do show that prophylaxis and therapy draw on the same shrinking pool of susceptibility.

### 2.4 Why surveillance cannot see it

National consumption reporting and the WHO GLASS-AMC platform aggregate ceftriaxone as a single line of defined daily doses (DDDs) procured or dispensed (World Health Organization, 2020). DDDs quantify volume, not indication, so the share generated by surgical prophylaxis cannot be recovered from them. Facility prevalence surveys do capture indication. One western Kenyan referral hospital identified ceftriaxone in 36.7% of prescriptions, much of it for prophylaxis of excessive duration (Sifuna et al., 2026), but these are episodic audits whose signal never reaches the national totals governing procurement. The AWaRe framework sets targets for reducing Watch consumption yet cannot reveal that a share of it is used needlessly in theatre (Zanichelli et al., 2023).

National Action Plans carry the same blindness: they commit to stewardship and surveillance, but none specifies an indication-stratified indicator for prophylaxis appropriateness, and stewardship teams, where they exist, rarely examine theatre practice.

The consequence is that the exposure cannot currently be quantified at the same level as overall consumption or resistance. *E. coli* and *K. pneumoniae* are GLASS priority pathogens whose 3GC resistance is reported centrally, but that denominator is built from GLASS priority specimens, namely, blood, urine, faeces, and genital swabs, rather than from surgical-site or wound specimens, where resistance runs higher; and GLASS-AMR sits in a separate module from GLASS-AMCand is linked to consumption only ecologically and never to indication (World Health Organization, 2020). Thus, current surveillance provides limited capacity to link ceftriaxone use in surgical prophylaxis with resistance outcomes. Without routine measurement, the extent of the practice cannot be reliably benchmarked or incorporated into stewardship and procurement decisions.

## 3 Discussion

Ceftriaxone-by-default is conventionally framed as prescriber behaviour to be corrected by education. This framing may be incomplete. The practice persists across health systems with different training, guidelines, and case mixes and may also reflect institutional and supply-chain factors, including availability of alternative agents and prescribing routines. Each is a structural lever involving an actor other than the surgeon.

The instruments in use, chiefly the Global Point Prevalence Survey, the ESAC protocols, and national quality-indicator sets, record duration, documented indication, and guideline concordance (**Table 2**) (Versporten et al., 2018). Each was built to measure whether prophylaxis was administered correctly; none to measure what it cost. A system can report 95% compliance while ceftriaxone dominates the non-compliant remainder, and no established indicator would reveal it. The gap is not uniformly one of absent instruments. For agent choice, the data are already collected: point-prevalence protocols record the molecule, the indication, and the duration in one record, and AWaRe-based quality indicators now cover surgical prophylaxis explicitly (Funiciello et al., 2024). What does not happen is transmission. Those figures live in episodic, facility-level surveys and never enter the national consumption totals that govern procurement or the indicators in National Action Plans. We propose four measures: one needing routing rather than building and three genuinely absent (**Table 2**).

**Table 2:** What surgical prophylaxis is already measured for, what is measured but not transmitted, and what is absent.

Measure	Failure mode captured	Status	Data source
Days of therapy per episode; percentage continued beyond 24 h	Prolongation	Established (Global-PPS, ESAC, national quality-indicator sets)	Theatre register; drug charts
Percentage of episodes carrying a documented indication	Empirical spillover	Established (Global-PPS, ESAC, national quality-indicator sets)	Prescribing records
Percentage of prophylactic episodes using a Watch agent rather than cefazolin	Substitution: agent identity	Measure 1. Collected in point-prevalence surveys and AWaRe quality indicators, but not carried into national reporting	Theatre register; pharmacy; PPS export
Cefazolin stock-out days, recorded alongside prescribing	Substitution: supply constraint	Measure 2. Absent from existing sets	Procurement records
Ceftriaxone DDDs attributable to prophylaxis, disaggregated from therapeutic use	Cross-cutting exposure	Measure 3. Absent from existing sets	Theatre register and prescribing audit, reconciled with AMC
Peri-operative carriage of 3GC-resistant Enterobacterales (sentinel sites)	Cross-cutting ecological outcome	Measure 4. Absent from existing sets	Rectal-swab surveillance; microbiology
Percentage of episodes with ceftriaxone plus metronidazole and no documented anaerobic indication	Combination	Partially captured (guideline concordance only)	Drug charts

Global-PPS, Global Point Prevalence Survey of Antimicrobial Consumption and Resistance; ESAC, European Surveillance of Antimicrobial Consumption; DDD, defined daily dose; AMC, antimicrobial consumption; 3GC, third-generation cephalosporin.

Measure 1. Agent share (substitution). This measure is the proportion of prophylactic episodes using a Watch-group agent rather than cefazolin. The numerator would be the number of eligible prophylactic episodes receiving a Watch-group agent without a documented procedure-specific justification, allergy, contraindication, or other guideline-supported alternative; the denominator would be all eligible surgical prophylaxis episodes assessed during the reporting period. This is derivable today from point-prevalence data and already formalised within AWaRe-based quality indicators (Funiciello et al., 2024), but it is reported facility by facility rather than nationally. Carrying it into routine national reporting is the smallest of the four proposed measures and the prerequisite for the others. One caution applies: where an indicator scores guideline concordance rather than the identity of the molecule, a formulary in which ceftriaxone is the concordant choice scores well while exerting the greatest selection pressure.

Measure 2. Cefazolin stock-out days (substitution: the supply constraint). This measure would be recorded alongside prescribing. This would be defined as the number of facility-days during which cefazolin was unavailable when required; when expressed as a proportion, the denominator would be the total monitored facility-days during the reporting period. No antimicrobial resistance (AMR) indicator set pairs a procurement measure with a prescribing measure, yet a guideline recommending an agent that cannot be ordered is inert. Procurement is a stewardship intervention; where shortages occur, measurement would help distinguish supply-related substitution from prescribing preference.

Measure 3. Prophylaxis-attributable ceftriaxone consumption. This measure is defined as the ceftriaxone doses administered for surgical prophylaxis during the reporting period, identified from theatre and prescribing records and converted to DDD equivalents using the applicable WHO DDD. These estimates would be compared with pharmacy dispensing records and national/GLASS-AMC consumption totals, while documenting differences between prescribed, dispensed, and administered quantities. Prevalence surveys yield proportions of prescriptions, not doses, and nothing reconciles indication-stratified prophylactic exposure with consumption totals. This is the central omission and the reason why no stewardship intervention in theatre can demonstrate an effect on national consumption. The barrier is an absent variable, not an absent system: a mandatory indication field where prescribing is electronic, a line on the chart where it is not.

Measure 4. Peri-operative carriage of 3GC-resistant Enterobacterales. This is the direct measure of the selection that prophylactic choice imposes on a patient's own flora, and the only one of the four denominated in resistance rather than in adherence or infection. The numerator would be participants with 3GC-resistant Enterobacterales detected in peri-operative surveillance samples, with the denominator being participants with valid samples collected at the specified peri-operative time point. It is the most demanding of the four; sentinel sites with existing microbiology capacity suffice. This measure is intended primarily as a research and sentinel surveillance component rather than routine clinical screening and would require evaluation of feasibility, cost, workload, and clinical implications before wider implementation. Such surveillance would allow the hypothesised relationship between prophylactic exposure and peri-operative carriage to be evaluated empirically rather than inferred from pharmacology and clinical isolates. Interpretation would require consideration of baseline colonisation, other antibiotic exposures, and potential hospital transmission, and colonisation findings should not independently prompt escalation to broader-spectrum prophylaxis.

One caution should accompany these measures. High resistance among wound isolates must not be read as an argument against cefazolin. The same pooled data show resistance to first- and second-generation cephalosporins scarcely lower than to third-generation agents (Monk et al., 2024), and every gram-negative isolate in the Rwandan series was cefazolin-resistant (Velin et al., 2021). These figures describe organisms cultured from infections that occurred, the prophylactic failures, rather than the flora of patients in whom prophylaxis succeeded. They therefore cannot by themselves establish which prophylactic agent would provide the greatest clinical benefit in each surgical population. The pooled review's own sensitivity analysis, comparing prospective cohorts with cross-sectional studies more likely to capture treatment failures, found similar patterns between the two, so the extent to which prophylactic selection contributes to these resistance patterns remains uncertain.

Prophylaxis for clean and clean-contaminated surgery targets skin flora, principally staphylococci, for which cefazolin is selected rather than for gram-negative breadth. The live debate over broader cover in caesarean prophylaxis concerns adjunctive azithromycin: in the C/SOAP trial, a single dose added to cefazolin roughly halved the composite postoperative infection outcome, 6.1% versus 12.0% (Tita et al., 2016). That debate turns on what to add to the cephalosporin base, not which cephalosporin to use, and its base was cefazolin. Where ceftriaxone resistance among the relevant organisms is already high, its broader spectrum does not necessarily provide an advantage for routine prophylaxis. The rationale for cefazolin is therefore based on guideline-supported efficacy and narrower antimicrobial exposure, rather than on an assumption that all surgical isolates remain susceptible to it.

Reform is supported by existing quality-improvement experience rather than requiring entirely new infrastructure. At Thika Level 5 Hospital in Kenya, a locally developed policy advising preoperative prophylaxis and discouraging extended postoperative courses was audited with feedback to prescribers; from near-exclusive postoperative use, adoption of preoperative prophylaxis was rapid (Aiken et al., 2013; Sefah et al., 2025). This example suggests that defined recommendations, local ownership, and feedback can improve prophylaxis practice.

Comparability must be built in from the outset: an infection recorded under five names with inconsistent case definitions cannot be pooled across borders, so any indicator set must specify the case definition alongside the metric. The picture assembled here rests substantially on Kenya, Uganda, Tanzania, and Rwanda; South Sudan contributes no surgical resistance data; Burundi and the Democratic Republic of the Congo contribute only uncultured incidence data (Chu et al., 2015), and Somalia contributes isolates not disaggregated by specimen source (Ibrahim et al., 2025). This uneven evidence base limits the extent to which the findings can be generalised across the EAC. Adoption can be tiered: the first three measures are feasible wherever surgery is documented, while the fourth should initially be implemented at sentinel sites. The first three measures could therefore provide a practical starting point for generating comparable data, while sentinel surveillance could subsequently evaluate the relationship between prophylactic exposure and resistance.

## 4 Conclusion

The decision this argument calls for is administrative rather than clinical. Ministries of health, national AMR secretariats, and the Community's regional machinery already collect prescribing, procurement, and antimicrobial consumption data. The main gap is the lack of a standardised system that links indication, agent selection, and consumption across facilities and countries. Establishing such measures would allow ceftriaxone use for surgical prophylaxis to be quantified, compared across settings, and incorporated into stewardship and procurement decisions. Further surveillance is also needed to determine the contribution of prophylactic exposure to resistance and to identify the settings in which alternative prophylactic strategies would be most appropriate.

## Data Availability

The original contributions presented in the study are included in the article/supplementary material, further inquiries can be directed to the corresponding authors.
